# Evidence for better microphytobenthos dynamics in mixed sand/mud zones than in pure sand or mud intertidal flats (Seine estuary, Normandy, France)

**DOI:** 10.1371/journal.pone.0237211

**Published:** 2020-08-06

**Authors:** Jérôme Morelle, Pascal Claquin, Francis Orvain

**Affiliations:** 1 BOREA—Biologie des Organismes et Ecosystèmes Aquatiques (FRE CNRS-2030, IRD-207, MNHN, Sorbonne Université, UA, UniCaen), Caen, France; 2 Normandie Université, Université de Caen Normandie, Caen, France; Centro de Investigacion Cientifica y de Educacion Superior de Ensenada Division de Fisica Aplicada, MEXICO

## Abstract

Understanding the dynamics of microphytobenthos biomass and photosynthetic performances in intertidal ecosystems will help advance our understanding of how trophic networks function in order to optimize ecological management and restoration projects. The main objective of this study was to investigate microphytobenthic biomass and photosynthetic performances as a function of the sedimentary and environmental variabilities in the range of intertidal habitats in the downstream Seine estuary (Normandy, France). Our results highlight higher biomass associated with more stratified biofilms and better photosynthetic performances in areas characterized by a sand/mud mixture (40–60% of mud) compared to pure sand or pure mud environments. This type of sediment probably offers an efficient trade-off between the favorable characteristics of the two types of sediments (sand and mud) with respect to light penetration and nutrient accessibility. Moreover, the large quantities of exopolysaccharides produced in sand/mud mixtures emphasizes the functional role played by microphytobenthos in promoting sediment stability against erosion. This allows us to show that despite the strong increase in sand content of the downstream Seine estuary, intertidal flats are still productive since microphytobenthic biomass, photosynthetic performances and exopolysaccharides secretion are highest in sand-mud mixtures. This study also underlines the impact of ecosystem modifications due to human disturbance and climate change on the dynamics of key primary producers in estuaries.

## Introduction

Microphytobenthos (MPB) is an assemblage of photosynthetic microalgae and cyanobacteria that form biofilms in intertidal and subtidal areas [1]. In areas with cohesive sediment, biofilms are typically composed of epipelic diatoms able to migrate vertically to access nutrient supplies and let light penetrate the sediment [2]. In contrast, epipsammic diatoms live attached to sediment grains in areas characterized by higher hydrodynamic stress and intrinsically mobile sands, where MPB diversity and biomass stocks are lower [3]. MPB is a key food source for local deposit-feeders that themselves support the upper levels of the trophic web such as birds and fish [4], but after resuspension, is also a food source for local suspension-feeders [5–8], particularly in turbid shallow water systems [8–10]. Therefore, MPB constitutes a large pool of photosynthetically competent organisms that significantly contribute to autochthonous primary production [11–13]. Consequently, understanding the spatial and temporal dynamics of intertidal MPB in relation with biological and environmental variability is crucial to understand coastal ecosystems, and their restoration and management.

While the distribution of biomass by MPB has frequently been studied in large scale ecosystems like estuaries or bays [6,14–18], photosynthetic performances of benthic diatoms have rarely been investigated at that scale [19]. In estuarine systems, the seasonal dynamics of MPB appears to follow the same trend every year with low inter-annual variability [17]. This typical pattern includes peaks of biomass in spring and autumn [17]. In the literature, there is a general consensus that microphytobenthic biomass and production vary as a function of many multifactorial effects including temperature [20], irradiance [21,22], nutrient concentrations, sediment disturbances, wind-induced resuspension [23], bioturbation [24], tidal variation [25], and grazing pressure by benthic fauna especially in summer [26–30]. Moreover, interactions between biological and physiological processes can be strongly affected by anthropogenic modification of natural ecosystems [31]. Despite these multifactorial effects, the main temporal changes that regulate MPB production rates are seasonal, driven by temperature effects [20,32] but also by spatial and temporal gradients in light availability, particularly because intertidal sites are subject to diel illumination mediated by tidal immersion [1], and to steep vertical irradiance gradients in the sediment [33] that differ with spatial grain size patterns. Consequently, despite the fact that chlorophyll *a* contents can be measured down to a depth of several centimeters, euphotic area is highly limited in the uppermost millimeters [34–37]. To cope with the large amount of light than can occur at the surface of intertidal mudflats during emersion (up to 2,000 μmol photons m^-2^ s^-1^), epipelic diatoms migrate vertically using exopolysaccharide secretions [38] to optimize photosynthesis [2,21,39,40] and avoid degradation of their photosystems due to excess light exposure [21,22,41]. Consequently, different vertical organization of MPB biomass, which also depends on sediment grain size [42], hydrodynamic mixing and bioturbation [43,44], needs to be carefully assessed when investigating MPB activity in response to physical disturbances or to predation [45,46].

Due to the importance of microphytobenthos in pools of autochthonous primary producers in estuarine systems, the main objective of this study was to estimate its spatial dynamics in the Seine estuary (Normandy, France) [47] which has never been previously investigated in this major ecosystem. Linked to one of the largest urban centers in Europe (Paris and a 67,000 km^2^ watershed), the Seine estuary is a highly dynamic ecosystem in terms of physical forcing [48,49] and is exposed to high human disturbance [50,51]. The three mudflats occurring along the salinity gradient of the downstream estuary were investigated in two contrasted months (September and April) to estimate the biomass and the photosynthetic performance dynamics of the microphytobenthic compartment. Our sampling design allowed us to consider several habitat features (from pure sand to pure mud) and several gradients (from the upper to the lower foreshore, from upstream to downstream zones).

## Material and methods

For each mudflat studied, all the samples were taken with the agreement and in the presence of a representative of the Reserve "maison de l'estuaire", Thomas Lecarpentier, who was our contact throughout the project. No further authorization was required, and this field study did not involve endangered or protected species.

### Study site and sampling

**Fig 1:**
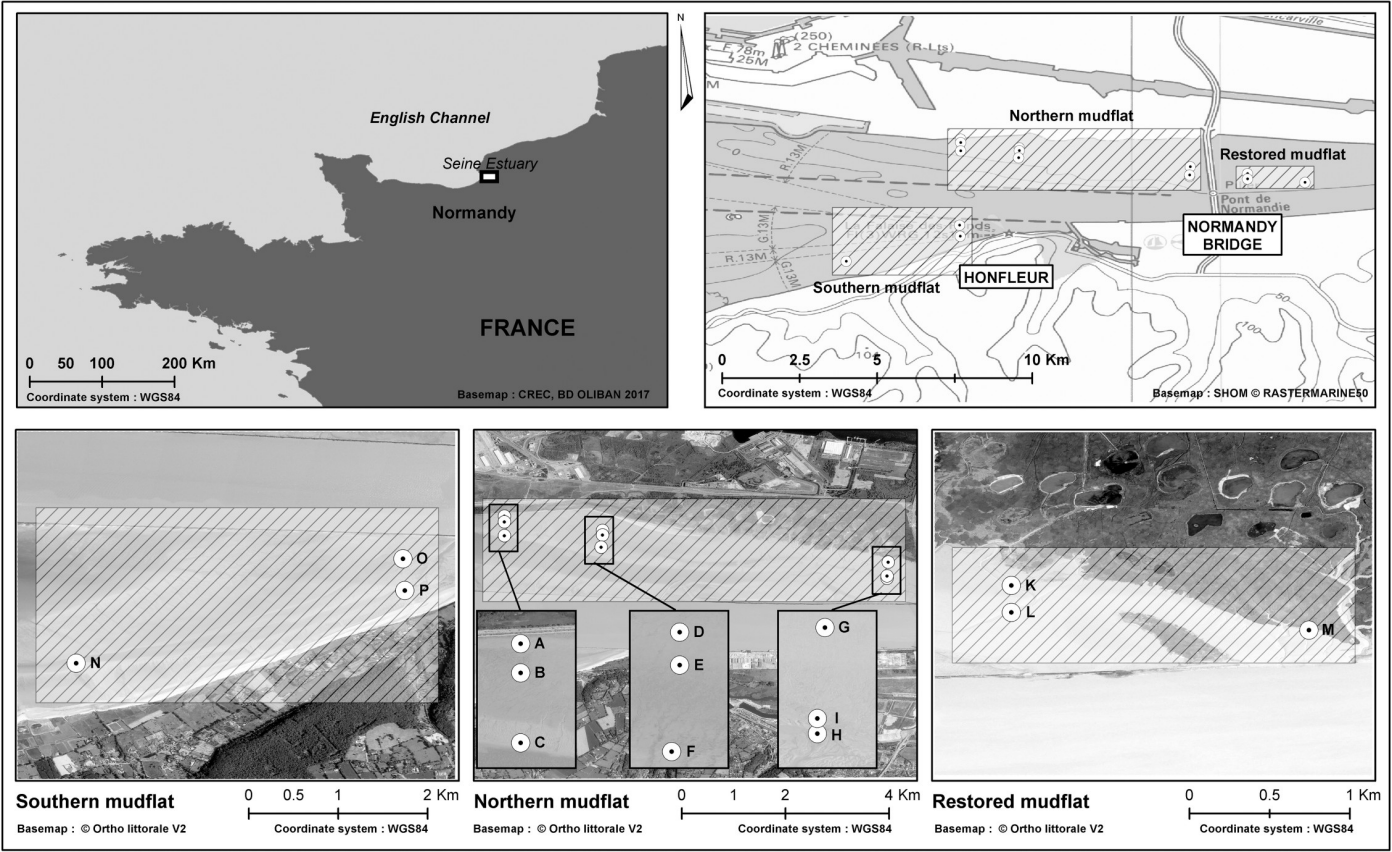
Location of the Seine estuary (Normandy, English Channel, France). The 15 sampling sites in the three respective areas: (1) the northern mudflat (three radials: A, B, C—D, E, F—G, H, I), (ii) the restored mudflat (K, L, & M), and (iii) the southern mudflat (N, O, & P).

Sampling was conducted on the three mudflats occurring along the salinity gradient of the downstream estuary of the Seine River (Fig 1). The three mudflats are characterized by different sediment structure: the southern mudflat is mostly sandy, the northern mudflat is a mixture of sand and mud, and the restored mudflat (corresponding to an artificially modified area upstream from the “Pont de Normandie” restored after extension of the port when the site became a Nature National Reserve) is basically muddy. Fifteen sites were studied successively across these tidal flats (Fig 1) in September 2014 and April 2015.

**Fig 2:**
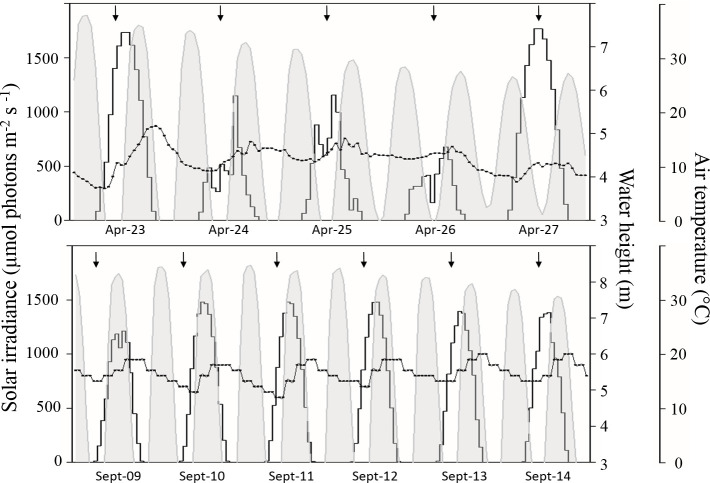
Variations in the environmental parameters during the course of the surveys. April sampling is shown at the top and September sampling at the bottom. The histograms represent solar irradiance (μmol photons m-2 s-1), the gray areas represent water height (m) and the line linking the square symbols represents air temperatures (°C). The black arrows represent the diurnal emersion period during which samples were collected.

During the surveys, solar irradiance varied cyclically up to 1,482 μmol photons m^-2^ s^- 1^ in September and up to 1,768 μmol photons m^-2^ s^-1^ in April. Temperatures were relatively constant during each campaign, average 16.5 ± 1.9°C in September and 11.2 ± 2.4°C in April (Fig 2). The sites were sampled successively throughout the week during the diurnal emersion period and all the parameters were measured in a 1 x 1 m square randomly selected at each site. Before core sampling, photosynthetic performances were measured by performing rapid light curves using a fiber-PAM fluorometer in three random locations inside the square. Inside the square, three cores (20 cm diameter × 1 cm deep) were sampled to determine grain size, water content, volumetric mass, dry bulk density, sediment specific attenuation of light coefficient, and biological parameters, i.e. exopolysaccharides, organic matter, chlorophyll *a*, and pheopigment contents. After being carefully homogenized, the sediment was split into flasks using cut syringes (to control the volume of sediment sampled) and stored at -20°C until analysis of each parameter. To determine the vertical distribution of chlorophyll *a*, three mini-cores (1.2 cm in diameter down to a depth of 2 cm) were sampled very close to the PAM measurement locations. The mini-cores were immediately frozen in the field using liquid nitrogen vapor, transported to the laboratory, and stored at –80°C until analysis.

### Sedimentary parameters

At each site, 20 ml of fresh sediment obtained from the cores (20 x 1 cm) was weighed in three replicates to calculate the volumetric mass values (in kg L^-1^). Each replicate was then dried in an oven at 60°C for three days and water content (ω; %) was determined as the percentage of water lost relative to total dry weight. The dry bulk density (C_sed_, kg m^-3^) was estimated using water content (ω; %) and grain density (*γ_s_*; kg m^-3^) according to Eq (1).

$$C_{sed}=\frac{\left(\gamma_{s}\times 1000\right)}{\frac{\omega }{100}\times \gamma_{s}+1000}$$ (1)

To determine the particle grain size, 5 ml of sediment from the cores (20 x 1 cm) were digested in 6% hydrogen peroxide for 48 h to remove organic matter. The grain size distribution was then measured on sub-samples using a LS Coulter particle size analyzer. The mud sediment fraction (i.e. mud content) was estimated as the percentage of silt particles < 63 μm (% fine) and the median grain size diameter was estimated from the cumulative percentage histogram.

The light attenuation coefficient in the sediment (k*_d(sed)_ in mm^-1^) was determined for the 15 sites sampled in September 2014 in three replicates per site. In multi-well plates, 9 wells per replicate containing 200 μl of milliQ water were cautiously filled with dry sediment from the cores (20 x 1 cm) to obtain different sediment thicknesses (25, 50, 75, 100, 125, 150, 200 and 400 μm). To obtain these thicknesses, a weight (mg) was determined for each well considering the volume of the well (cm^3^) and the measured dry bulk density (C_sed_; g cm^-3^). The exact thickness was then corrected as a function of the measured weight. After the sediment settled homogeneously at the bottom of the well, light absorbance was read at the same wavelength as the fiber-PAM fluorometer (460 nm; cf. 2.3.3) using a FlexStation™ fluorescent microplate reader (Molecular Devices, Sunnyvale, CA, USA). Thereafter, k*_d(sed)_ was determined for each sample using an exponential regression on the absorbance curves against thickness according to Eq 2 where k*_d(sed)_ is represented by the coefficient *a*.

$$y=e^{-ax}$$ (2)

### Biological parameters

#### Chlorophyll *a* and pheopigment contents

To calculate the chlorophyll *a* content (chl *a* in μg gDW^**-1**^), three replicates of 1.5 ml fresh sediment from cores (20 x 1 cm) were sampled and lyophilized. Then, 1 g of sediment from each replicate was weighed and pigments were extracted in 10 ml of 90% acetone for 18 h under continuous mixing by automatic rotation in the dark at 4°C. After centrifugation (4°C, 3,000 rpm, 10 min), the fluorescence of the supernatant was measured using a Turner Trilogy fluorometer (Turner Designs, Sunnyvale, California, USA) before and after acidification (10 μl of HCl, 0.3 M per 1 ml of sample). Chl *a* values (in μg gDW^**-1**^) and pheopigment contents were then calculated using the [52] and converted into mg m^**-2**^ using the dry density of the sediment considering a sample depth of 1 cm [53]. The pheopigment contents were transformed into a percentage by calculating the following ratio:

[Pheocontent]/([Pheocontent+Chlacontent])×100.

#### Biomass vertical profiles

To examine the vertical distribution of chlorophyll *a*, the sampled mini-cores were sliced using a freezing microtome (-25°C) in the two weeks following sampling. Eleven depth intervals were sliced: 0–200; 200–400; 400–600; 600–800; 800–1,000; 1,800–2,000; 2,800–3,000; 3,800–4,000; 5,800–6,000; 7,800–8,000 and 9,800–10,000 μm. Each sliced section was placed in a pre-weighed Eppendorf tube and freeze-dried. The dry mass was measured and fluorescence of chl *a* was measured using a TurnerTD-700 fluorimeter according to the method of [54].

To compare stratification of the biofilm on the chl *a* depth profiles, a biofilm structure index (BSI) was calculated by dividing the mean value of chl *a* in the top layer (0 – 1,000 μm) by the mean value in the underlying layer (1,000–10,000 μm). Thereby, BSI > 2 was characteristic of a stratified biofilm (significantly higher chl *a* concentration in the top layers) and a BSI < 2 was more characteristic of a homogeneous profile.

#### Photosynthetic parameters

Fluorescence was measured in triplicate at each site using a Fiber-PAM fluorometer (PAM-control unit and WATER-EDF-universal emitter detector unit; Walz, Effeltrich, Germany). The distance between the tip of the fiber optic probe and the surface of the sediment was kept constant at 2 mm for each measurement using a burette holder whose base was buried in the sediment. A 4-cm diameter dark circular adaptor (supplied with the device) fixed around the optic fiber isolated the sample from natural light and allowed us to control the length of dark adaptation before measurement. The background signal was measured in the deep layer of sediment (20–30 cm) at the site in the absence of active photosynthetic cells and was automatically subtracted from fluorescence values.

After 5 min dark adaptation, which is a compromise between oxidation of the Quinone pool and vertical migration of microphytobenthic cells at depth [55], rapid light curves (RLC) were performed. During RLC, the sample was excited by a low frequency measuring light (1 μmol photons m^-2^ s^-1^, 470 nm, frequency 0.6 kHz) to determine the initial level of fluorescence (F_O_). Maximum fluorescence (F_M_) was then obtained using a saturating light pulse (0.6 s, > 10 000 μmol photons m^-2^ s^-1^, 460nm), allowing the Quinone A, Quinone B and part of plastoquinone pools to be reduced. The maximum effective quantum yield of the PSII [56] was then estimated as F_V_/F_M_ = (F_M_-F_O_)/F_M_. Subsequently, each replicate was exposed to nine actinic irradiance steps (E: 0 to 929 μmol photons m^-2^ s^-1^ in September 2014 and 0 to 2309 μmol photons m^-2^ s^-1^ in April 2015) for 30 seconds at each step. At each actinic light step, a steady state fluorescence (F_S_) and a new maximum fluorescence (F_M_’) were measured to enable calculation of the effective quantum yield of PSII (ΔF/F_M’_). Relative electron transport rates (relative units) were then calculated for each actinic light step (rETR = ΔF/F_M’_ x E).

To account for light and fluorescence attenuation in the sediment, the tool developed by Morelle *et al*. (2018a) was used to correct each RLC, considering the chl *a* depth profile, particle grain size and the light attenuation coefficient (k*_d(sed)_) obtained as previously described.

Maximum light efficiency (α; rel. unit) and relative maximum electron transport rate (rETR_max_; rel. unit) were estimated from corrected curves using the fit Eq 3 [57] and the light saturation intensity (E_k_; μmol photons. m^-2^. s^-1^) was calculated as E_k_ = rETR_max_/α.

$$\mathrm{rETR}={\mathrm{rETR}}_{\max}\times \left(1-e^{\frac{-\alpha }{\left(\mathrm{rETRmax}\times E\right)}}\right)$$ (3)

The thermal dissipation of excess absorbed light energy corresponding to non-photochemical quenching (NPQ) of fluorescence was also calculated as NPQ = (F_M_-F_M_’)/F_M_’ and the fit Eq 4 [58] was used to estimate the maximum NPQ value reached during the light curve (NPQ_max_) and the irradiance level for which NPQ attains 50% of NPQ_max_ (E_50_; μmol photons. m^-2^. s^-1^).

$$\mathrm{NPQ}\left(E\right)={\mathrm{NPQ}}_{\max}\times \frac{E^{n}}{E_{50}^{n}+E^{n}}$$ (4)

where *n* is the Hill coefficient, characterizing the sigmoidicity of the curve.

To compare the light responses of NPQ and ETR, the fraction of NPQ formed when ETR approaches saturation (i.e. when E = E_k_) was calculated using the indices E_50_/E_k_ and NPQ_Ek_ = NPQ(E_k_)/NPQ_max_ [58].

#### Exopolysaccharides

As rapidly as possible after sampling, exopolysaccharides (EPS) were extracted from 5 mL of fresh homogenized sediment placed in centrifugation tubes (15 mL) with 5 mL of artificial sea water. After 1 hour of incubation in a rotary shaker, the tubes were centrifuged at 3,000 rpm for 10 min at 4°C and the supernatants containing colloidal exopolysaccharides were collected and placed in a new centrifugation tube. The pellet was used to extract bound EPS with 5 mL of artificial seawater and ~1g of activated cationic resin (Dowex Marathon C, Na+; Sigma-Aldrich). After resuspension and one hour of incubation in a rotary shaker, the tubes were centrifuged at 3,000 rpm for 10 min at 4°C and the supernatants were collected. In both fractions, high and low molecular weight EPS (respectively HMW and LMW EPS) were separated by incubation of supernatants into ethanol (70% final concentration) for 16 hours at - 20°C. After centrifugation at 3,000 rpm for 30 min at 4°C, LMW EPS, present in the supernatant, were discarded while HMW EPS in the pellet were dried at 60°C in a dry bath under airflow for from 6 to 48 hours. Dried samples were suspended in 3 ml of milliQ water for carbohydrate and protein quantification. Carbohydrate contents were estimated using sulfuric acid and phenol with glucose as a standard [59]. Protein contents were estimated using the Bradford assay with bovine serum albumin (BSA) as standard [60].

#### Organic matter

The organic content in the sediment samples was estimated by calculating weight loss after calcination (450°C; 4 h) of the dry samples obtained after characterization of sedimentary parameters described above.

### Statistical analyses

All fluorescence data treatment was performed using MATLAB [46] and the statistical tests cited below were performed using SigmaPlot or R Software. Multiple regressions were performed to estimate which parameters influenced the dynamics of the biological parameters. Correlations between parameters were tested using Pearson correlation tests. After testing the application conditions (normality of residuals, homoscedasticity), ANOVA (AOV) was used (or the non-parametric Kruskal-Wallis test (KT) when conditions were invalid) to estimate significant differences between sites. A principal component analysis (PCA) was performed on the dataset using the “MissMDA” and “FactoMineR” packages in R Software.

## Results

### Sedimentary parameters

**Fig 3:**
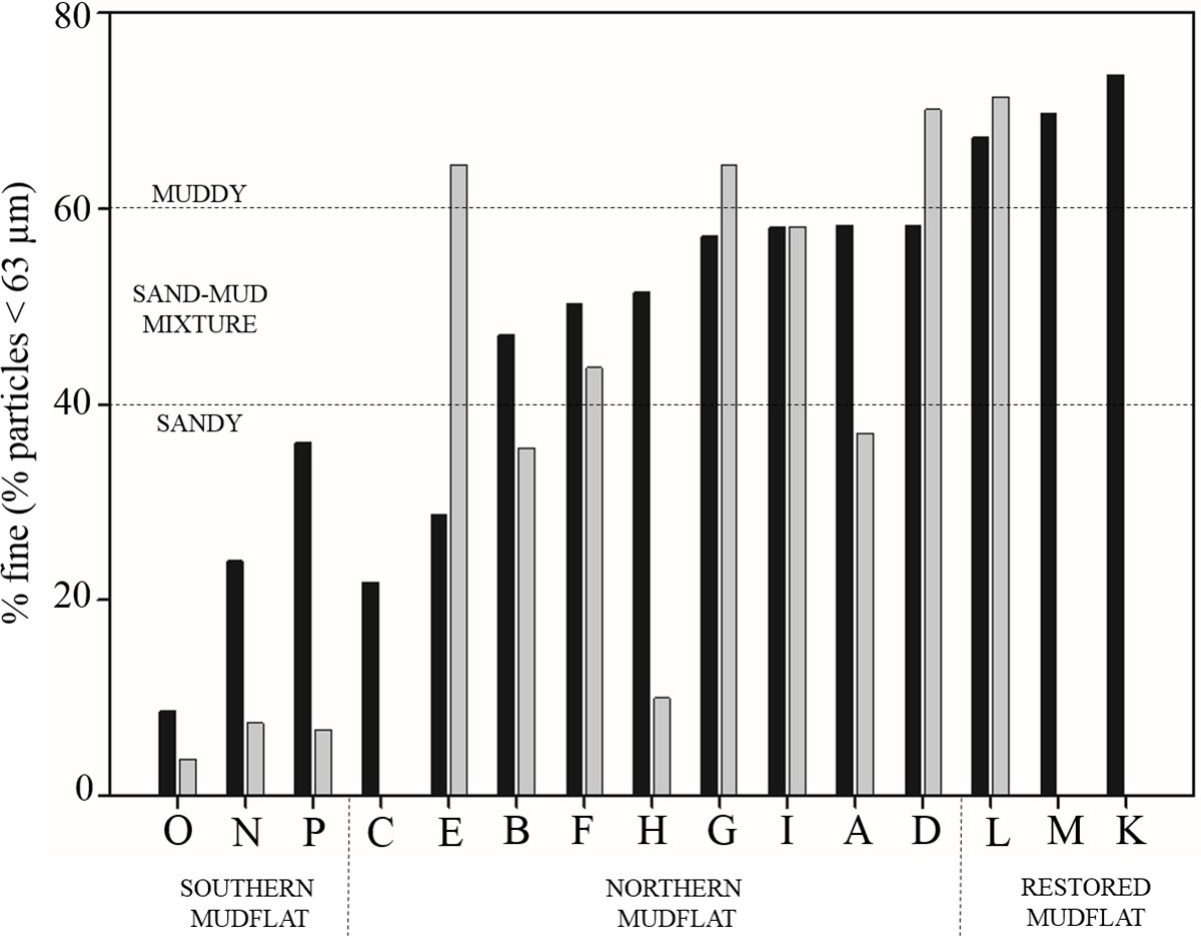
Histogram of grain size proportion in % fine (< 63 μm) at each sampling site. The black bars represent values in September and the gray bars in April. Sites were defined as sandy with < 40% mud content and as muddy with > 60% mud content.

The southern mudflat that included sites O, N and P (Fig 1), was characterized by sandy sediments with less than 36.1% of fine particles in September and less than 7.44% in April (Fig 3). This high sand content is mainly due to the high level of physical disturbance linked to the dominant current inside the estuary oriented NW [48,61]. Despite being located on the northern mudflat, sites C and E in September, and B and H in April can also be considered as sandy sites with less than 40% of fine particles. The pit located in the northern intertidal area could cause fine particles to be swept away by the strong flow thereby explaining the presence of these sandy areas. Sites K, L and M can be considered as muddy sites with more than 67.3% of fine particles in summer. Similarly, site L was characterized by 71.4% of fine mud in spring. All these sites are located in the restored mudflat, a protected area with low flow that is characterized by the accumulation of fine particles. All the other sites were considered as sand-mud mixtures with a percentage of fine particles ranging between 47.1% and 64.5%. Sites K, M and C were not sampled in April due to difficulty accessing the site and technical problems with the sampling equipment. The higher percentage of fine particles in September than in April at each site can be explained by calmer hydrodynamic conditions and reduced wind stress in summer. Indeed, the increase in the river flow in winter combined with the higher wave regime explain the decrease in fine particle content observed in spring especially at the lower limit of the foreshore.

**Fig 4:**
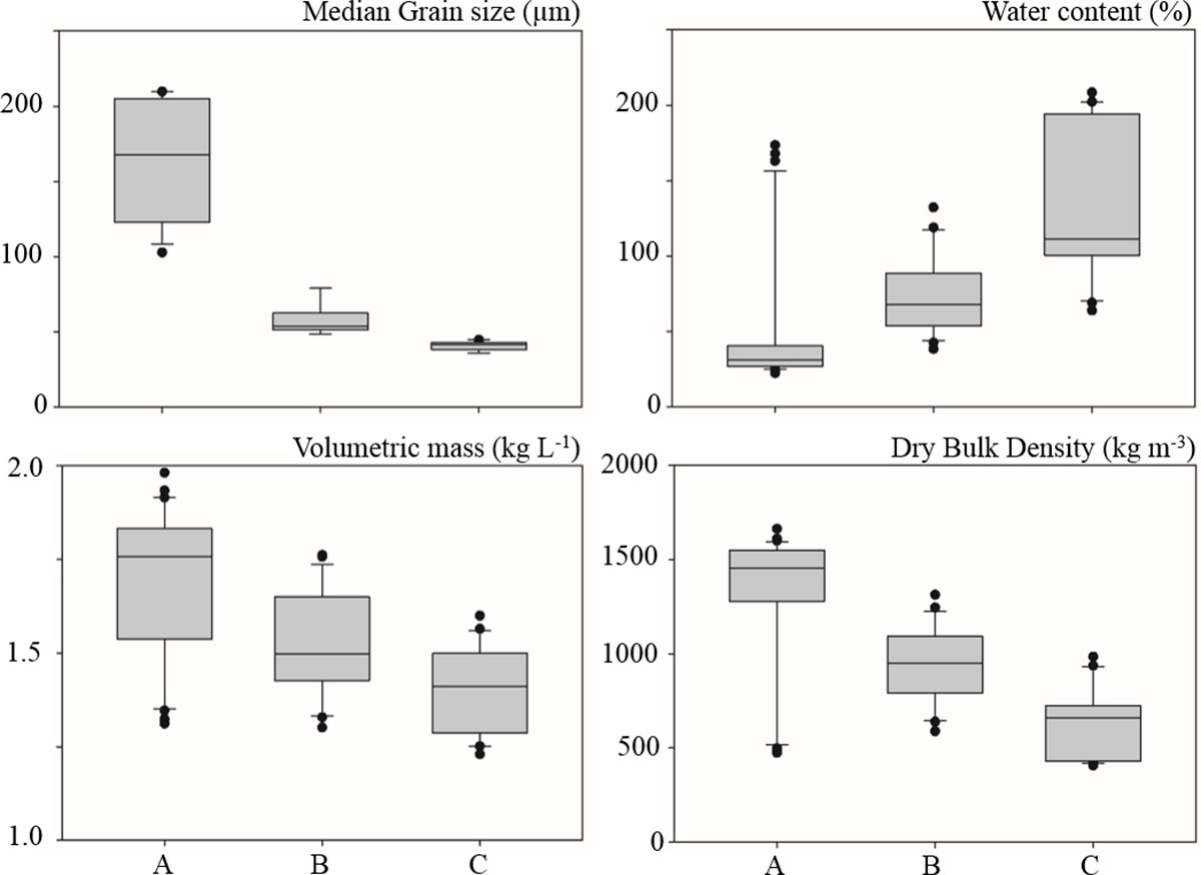
Boxplot of sedimentary parameters as a function of sediment type. Three classes were distinguished in both months: A. Sandy sites (% fine < 40%), B. Sand-mud mixture (%fine range between 40 and 60%) and C. Muddy sites (% fine > 60%).

As expected, the other sedimentary parameters were correlated with grain size. The correlation was high for water content (Pearson correlation: 0.64; p-value < 0.001) with low percentages (between 20% and 40%) at sandy sites, and high percentages (up to 200%) at muddy sites (Fig 4). Inversely, volumetric mass (Pearson correlation: -0.73; p- value < 0.001) and the dry bulk density (Pearson correlation: -0.78; p- value < 0.001) were higher at sandy sites (up to more than 1.8 kg L^-1^ and 1500 kg m^-3^ respectively) and lower in muddy areas (less than 1.3 kg L^-1^ and 500 kg m^-3^ respectively).

**Fig 5:**
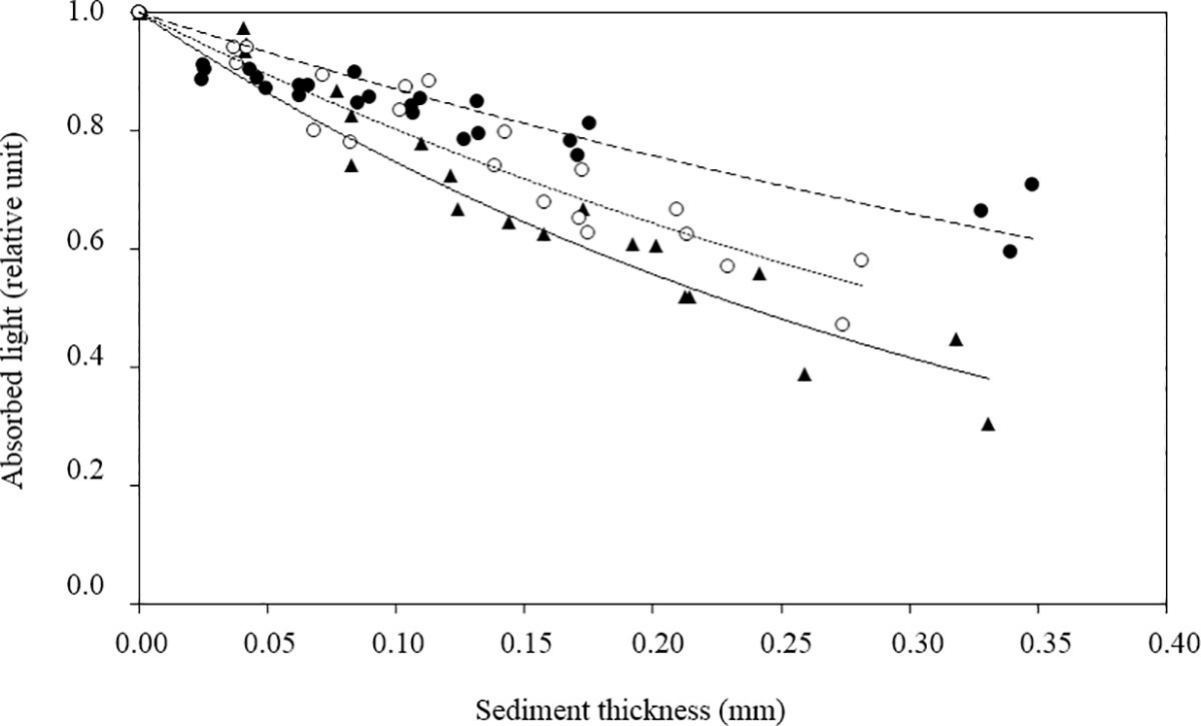
Absorbance of light as a function of sediment thickness and percentage of fine grained content enabled estimation of the light attenuation coefficient (k*d(sed); mm^-1^). Black triangles: sampling sites O (8.61%), white circles: H (51.50%); black circles: M (69.70%). k*_d(sed)_ is defined as the coefficient *a* of the equation y = e ^*a*x^. Here the coefficients obtained for O, H, and M were 2.35, 3.68, and 4.76 mm^-1^ respectively.

As explained by the deeper penetration of light in sandy than in muddy sediments, the attenuation coefficients of light (k*_d(sed)_) were inversely proportional to the median grain size in September (linear regression; R^2^ = 0.61; p < 0.001) according to the equation k*_d(sed)_ = - 0.0122 x Median + 4.89. Low values (< 3 mm^-1^) representative of slow light extinction were recorded at sandy sites. Values ranged from 3.32 to 5.36 mm^-1^ at the other sites indicating rapid and variable attenuation coefficients of light in intertidal areas where fine sediment particles accumulated (Fig 5; Table 1). As we were unable to measure the k*_d(sed)_ in April, the previous equation was used to estimate the spring values.

**Table 1:** Values of some photosynthesis related parameters estimated for each sampling site in September and April.

Sites	%fine	BSI	k*_d(sed)_	%Pheo	%OC	F_V_/F_M_	α	E_k_	NPQ_max_	E_50_	n	NPQ(E_k_)	rETR(E_k_)	E_50_/E_k_	NPQE_k_
**SEPTEMBER**															
**O**	8.61	0.9	2.35	38.85	0.66	0.62	0.55	622.21	15.80	> 5,000.00	0.89	0.45	220.47	38.89	0.03
**P**	21.80	7.6	2.75	78.38	1.20	0.57	0.50	676.11	10.40	> 5,000.00	1.19	0.15	213.87	28.50	0.01
**N**	24.00	2.0	5.02	76.26	0.18	0.71	0.62	799.78	39.83	> 5,000.00	2.26	0.13	334.73	11.35	0.00
**C**	21.80	7.6	2.75	78.38	0.75	0.57	0.50	676.11	10.40	> 5,000.00	1.19	0.15	213.87	28.50	0.01
**E**	24.00	2.0	5.02	76.26	1.14	0.71	0.62	799.78	39.83	> 5,000.00	2.26	0.13	334.73	11.35	0.00
**B**	47.10	2.9	4.28	51.02	1.47	0.58	0.53	816.46	8.76	1,179.73	18.45	0.14	313.83	1.44	0.02
**F**	50.30	2.4	4.15	60.00	1.61	0.60	0.53	880.04	2.65	2,706.81	0.80	0.49	317.03	3.08	0.18
**H**	51.50	1.6	3.68	79.13	0.52	0.61	0.61	1,173.75	0.50	617.08	5.63	0.56	800.70	0.53	1.12
**G**	57.20	1.7	3.43	57.32	0.40	0.60	0.56	819.80	0.91	987.05	48.71	0.00	295.67	1.20	0.00
**I**	58.10	1.6	3.87	66.70	0.37	0.57	0.55	798.55	68.55	1,431.31	15.02	0.00	303.03	1.79	0.00
**A**	58.30	2.8	4.93	52.84	1.54	0.57	0.47	997.44	NA	NA	1.06	1.00	295.50	NA	0.00
**D**	58.30	1.5	5.36	52.88	2.18	0.44	0.47	985.57	1.82	> 5,000.00	1.28	0.05	292.67	9.46	0.03
**L**	67.30	1.2	3.32	72.27	4.13	0.36	0.29	3,291.13	0.31	174.67	0.67	0.12	647.30	0.05	0.39
**M**	69.70	1.5	4.76	77.71	2.42	0.30	0.34	798.57	0.71	1,307.87	6.23	0.07	182.17	1.64	0.10
**K**	73.70	0.9	4.20	77.36	3.90	0.32	0.35	1,554.58	0.00	152.81	4.17	0.00	359.93	0.10	0
**APRIL**															
**O**	3.71	2.3	2.42	64.63	1.56	0.47	0.51	735.60	0.38	1,669.81	6.52	0.03	239.70	2.27	0.08
**P**	6.73	1.3	2.34	92.68	1.09	0.58	0.60	610.69	1.33	1,842.54	2.49	0.11	234.29	3.02	0.08
**N**	7.44	1.6	2.33	72.59	2.35	0.36	0.47	431.82	0.49	1,418.13	4.45	0.01	133.78	3.28	0.02
**H**	9.95	5.6	4.26	87.91	1.37	0.31	0.25	610.49	0.57	1,513.39	4.24	0.01	107.90	2.48	0.01
**B**	35.52	4.1	3.33	80.67	4.29	0.31	0.37	1,302.96	0.00	201.75	31.96	0.00	312.73	0.15	0.00
**A**	37.05	2.6	3.46	79.65	NA	0.52	0.48	1,291.27	0.00	162.69	91.65	0.01	412.10	0.13	-0.22
**F**	43.76	3.5	3.93	74.14	2.99	0.56	0.53	1,141.22	0.47	1,900.13	5.02	0.06	400.58	1.67	0.12
**I**	58.20	1.6	2.39	84.05	6.40	0.67	0.60	828.37	1.98	3,054.83	1.35	0.27	314.65	3.69	0.14
**G**	64.47	2.1	4.37	76.95	8.25	0.59	0.58	689.27	1.47	2,515.80	2.22	0.08	254.00	3.65	0.05
**E**	64.50	2.6	4.34	77.77	5.00	0.65	0.62	1,101.57	3.32	4,750.26	2.29	0.11	438.15	4.31	0.03
**D**	70.14	2.8	4.42	69.13	2.68	0.66	0.66	848.85	2.20	2,436.96	2.09	0.22	354.28	2.87	0.10
**L**	71.43	1.3	4.38	78.18	6.43	0.62	0.63	740.23	2.20	2,462.38	1.69	0.23	297.85	3.33	0.11

Percentage of fine content (%fine), biofilm stratification index (BSI), light attenuation coefficient in the sediment (k*_d(sed_) in mm^-1^), percentage of pheopigment (%pheo), and percentage of organic content (%OC). Effective quantum yield of the PSII (F_V_/F_M_; rel. unit), photosynthetic efficiency (α; rel.unit), and light saturation (E_K_ μmol photons m^-2^ s^-1^) obtained from RLC (rETR/E curves). Maximum thermal dissipation of excess-absorbed-light energy (NPQ_max_), irradiance level for which NPQ attains 50% of NPQ_max_ (E_50_; μmol photons. m^-2^. s^-1^), and the *n* parameter corresponding to the degree of sigmoidicity obtained from NPQ/E curves. The values of NPQ and rETR when rETR approaches saturation (i.e. when E = Ek) and the fraction of NPQ formed were calculated using indices E_50_/E_k_ and NPQE_k_ = NPQ(E_k_)/NPQ_max_.

### Biological parameters

#### Organic content

In both months, lower organic contents were recorded in the sandy area than in areas with a mixture of sand and mud and with pure mud (Table 1). In September, percentages were low in the southern and in the northern mudflats and no significant difference was observed between values (p- value = 0.23) ranging between 0.2% and 2.2%. The significantly (AOV; p-value < 0.001) highest percentages of organic contents were measured in the restored mudflat with values ranging between 2.4% and 4.1%. In April, the percentages were higher than in September with values ranging between 1.1% and 2.4% in the southern mudflat, between 1.4% and 8.3% in the northern mudflat and a value of 6.4% in the restored mudflat. Thereby, significantly lower values were measured in the southern mudflat than in the two others (AOV; p-value < 0.05) while no significant difference was observed between the northern and restored mudflats (AOV; p-value = 0.46).

#### Chlorophyll *a* content and distribution

**Fig 6:**
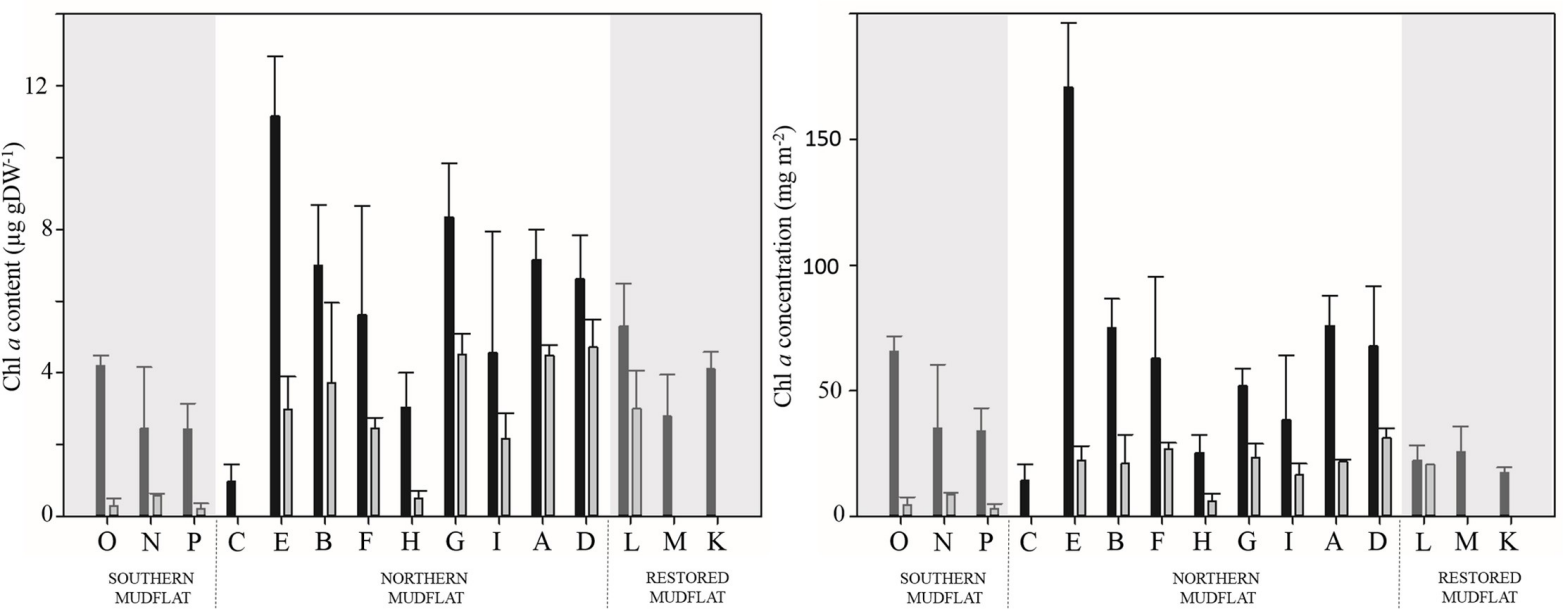
Histogram of chl *a* content (μg gDW^-1^; left) and concentration (mg m^-2^; right) for each sampling sites. Black bars: values for September samples, gray bars: values in April. Sites are ranked as a function of their percentage of fine particles (Fig 2) and their location on the different mudflats.

Chlorophyll *a* contents (chl *a;* μg gDW^**-1**^; Fig 6) were higher in September (ranging from 0.to 11.15 μg gDW^**-1**^) than in April (ranging from 0.22.to 4.72 μg gDW^**-1**^). In September, the highest chl *a* content was measured at site E: 11.15 μg gDW^**-1**^, and the lowest value at site C: 0.96 μg gDW^**-1**^. In April, the lowest value was measured at site P (0.22 μg gDW^**-1**^) and the highest at site D (4.72 μg gDW^**-1**^). The lowest values were always recorded at sites characterized by fine particle contents < 30%. Thus, the chl *a* values in the southern mudflat (sites O, P and N) were significantly lower than in the northern mudflats in both seasons (AOV; p- values < 0.05) with an average for the three sites of 3.02 μg gDW^**-1**^ in September and 0.36 μg gDW^**-1**^ in April. In the sites with more than 30% fine particle contents located in the northern mudflat and in the restored mudflat, no significant differences were found in either month between chl *a* content (AOV; p-values > 0.05) with an average of 5.46 ± 1.84 μg gDW^**-1**^ in September and 3.51 ± 0.99 μg gDW^**-1**^ in April. A negative relationship was found between pheopigment content (%) and chl *a* content (y = – 5.1 x chl *a* + 89, R^2^ = 0.61, N = 78; p-value < 0.001).

The chl *a* concentrations (mg m^-2^) also varied in space (Fig 6). In September, the lowest chl *a* content (14.17 mg m^-2^) was measured at site C and the highest (170.72 mg m^-2^) at Site E. In April, values ranged between 3.09 mg m^-2^ at site P and 31.06 mg m^-2^ at site D.

**Fig 7:**
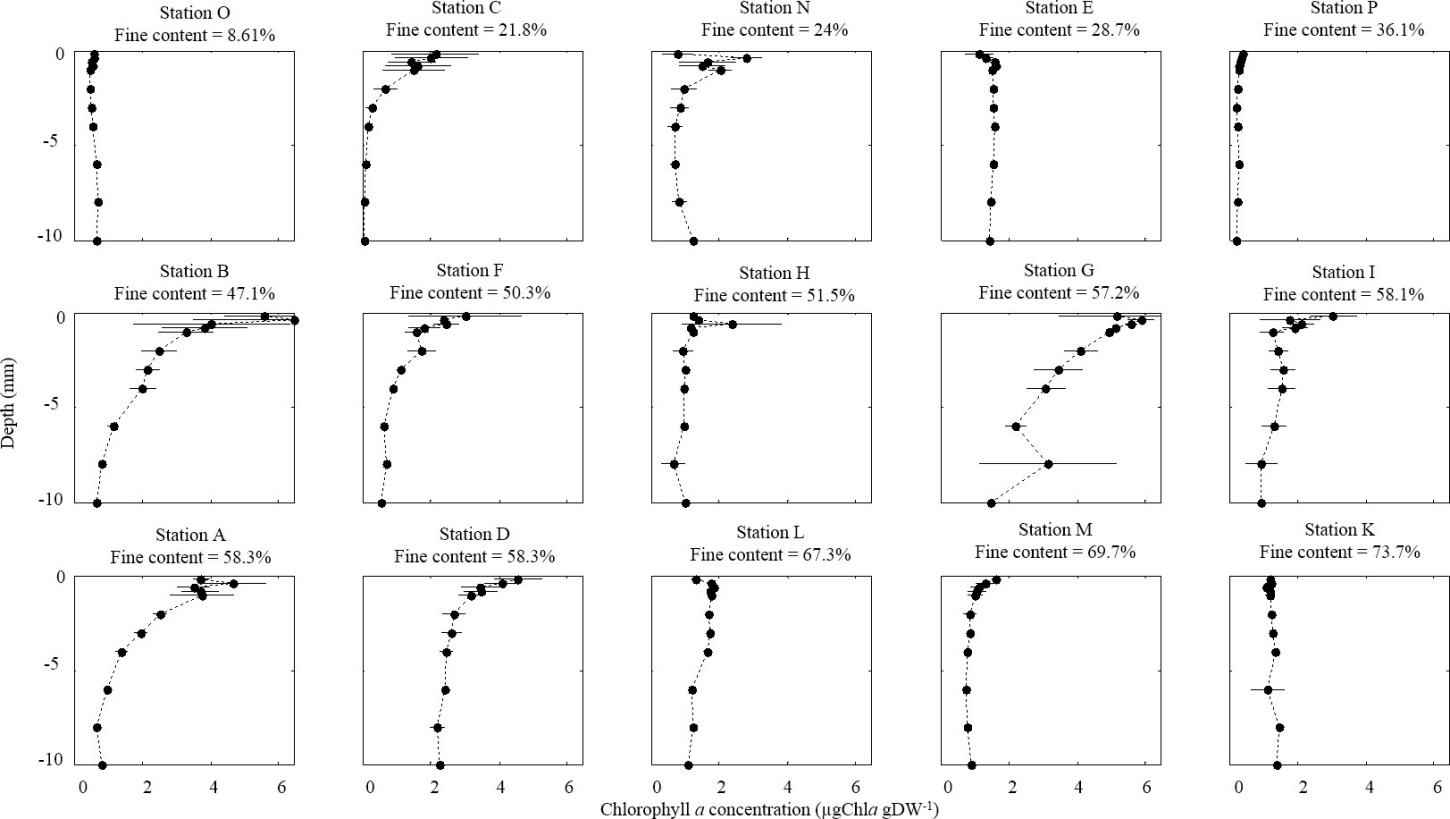
Vertical depth profile of chl *a* (μg gDW^-1^) at each sampling site in September 2014. Horizontal bars represent the standard deviation (n = 3).

In September, the chl *a* depth patterns varied notably among sites revealing marked variation in the MPB biofilm characteristics (Fig 7). Homogeneous profiles were observed at the sandiest site O (8.61% fine content (f.c.)) in the southern mudflat, and at the muddiest site K (73.7% f.c.) in the restored mudflat. Homogeneity was also observed at sites M (69.7% f.c.) and P (36.1% f.c.) but with slightly higher values in the surface layers than at depth; inversely, slightly lower values were observed in the surface layers at sites L (67.3% f.c.), H (51.5% f.c.) and E (28.7% f.c.). The other chl *a* profiles showed significantly higher values in the top layers (0–1 mm) than at depth (sites C, B, F, I and D). For sites N (24% f.c.), G (57.2% f.c.), and A (58.3% f.c.), higher values in the top layers (0 – 1 mm) than at depth were observed but with a lower value in the uppermost section than in other top layers (Fig 7).

**Fig 8:**
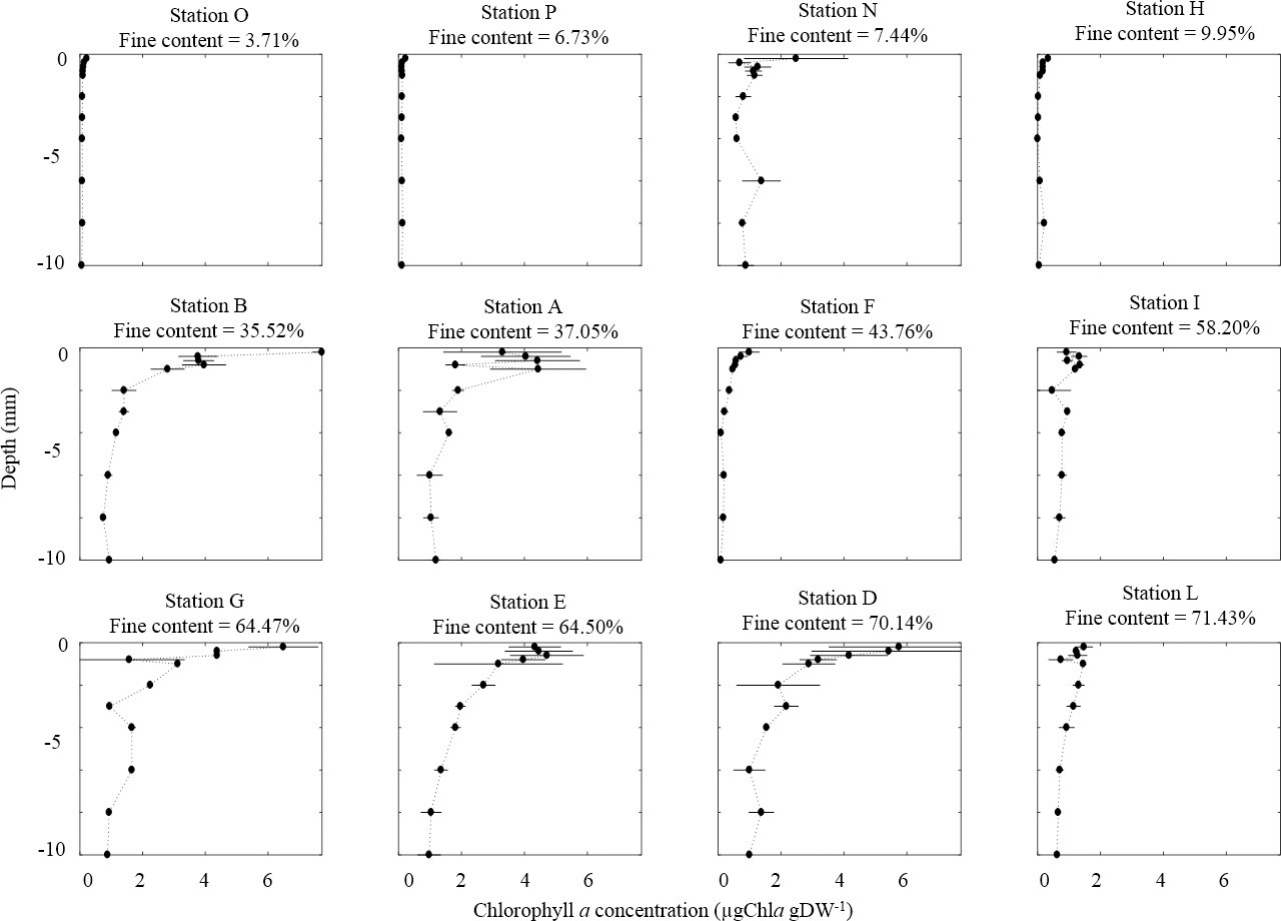
Vertical depth profile of chl *a* (μg gDW^-1^) at each sampling site in April 2015. Horizontal bars represent the standard deviation (n = 3).

In April (Fig 8), homogeneous profiles were observed at the muddiest site L (71.43% f.c.) in the restored mudflat, and at the sandiest sites O and P (3.71% and 6.73% f.c., respectively) in the southern mudflat. Vertical homogeneity was also observed at sites I (58.2% f.c.) and H (9.95% f.c.) in the northern mudflat. At sites E (64.5% f.c.) and A (37.05% f.c.), higher values were recorded in the top layers (0 – 1 mm) than at depth but with a lower value in the uppermost section than in other top layers (Fig 8). The other chl *a* profiles presented significantly higher values in the top layers (0–1 mm) than at depth (sites N, B, F, G and D).

The BSI values (Table 1) confirmed our observations (Figs 7 and 8) and allowed us to differentiate the homogeneous from the stratified vertical distribution of chl *a*. Thereby, we observed that established biofilms (BSI > 2) were mainly located recorded in the northern mudflat where the sediment was a mixture of sand and mud. Only sites O and H in April showed a high BSI value but a clear homogeneous profile, probably due to very low chl *a* concentrations along the profile.

#### Photosynthetic parameters

The maximum quantum yield of PSII (F_**V**_/F_**M**_) ranged from 0.30 to 0.71 with an average of 0.53 ± 0.11 in both months (Table 1). In September, the F_**V**_/F_**M**_ values were significantly lower (< 0.4) at muddy sites (K, L and M) than at the other sites (AOV; p-values < 0.001). In contrast, F_**V**_/F_**M**_ values in April were significantly lower (< 0.4) at several sandy sites (N, H and B) than at the other sites (AOV; p-values < 0.001).

The maximum light efficiency (α; rel.unit) ranged from 0.25 to 0.7 (Table 1). The lower values were observed in the restored mudflat in September with a mean value of 0.3 ± 0.1 while the other sites on the northern and southern mudflats had a mean value of 0.5 ± 0.05. In April, low values were observed at sites B and H (respectively 0.3 ± 0.03 and 0.4 ± 0.06) while the other sites had a mean value of 0.6 ± 0.05.

**Fig 9:**
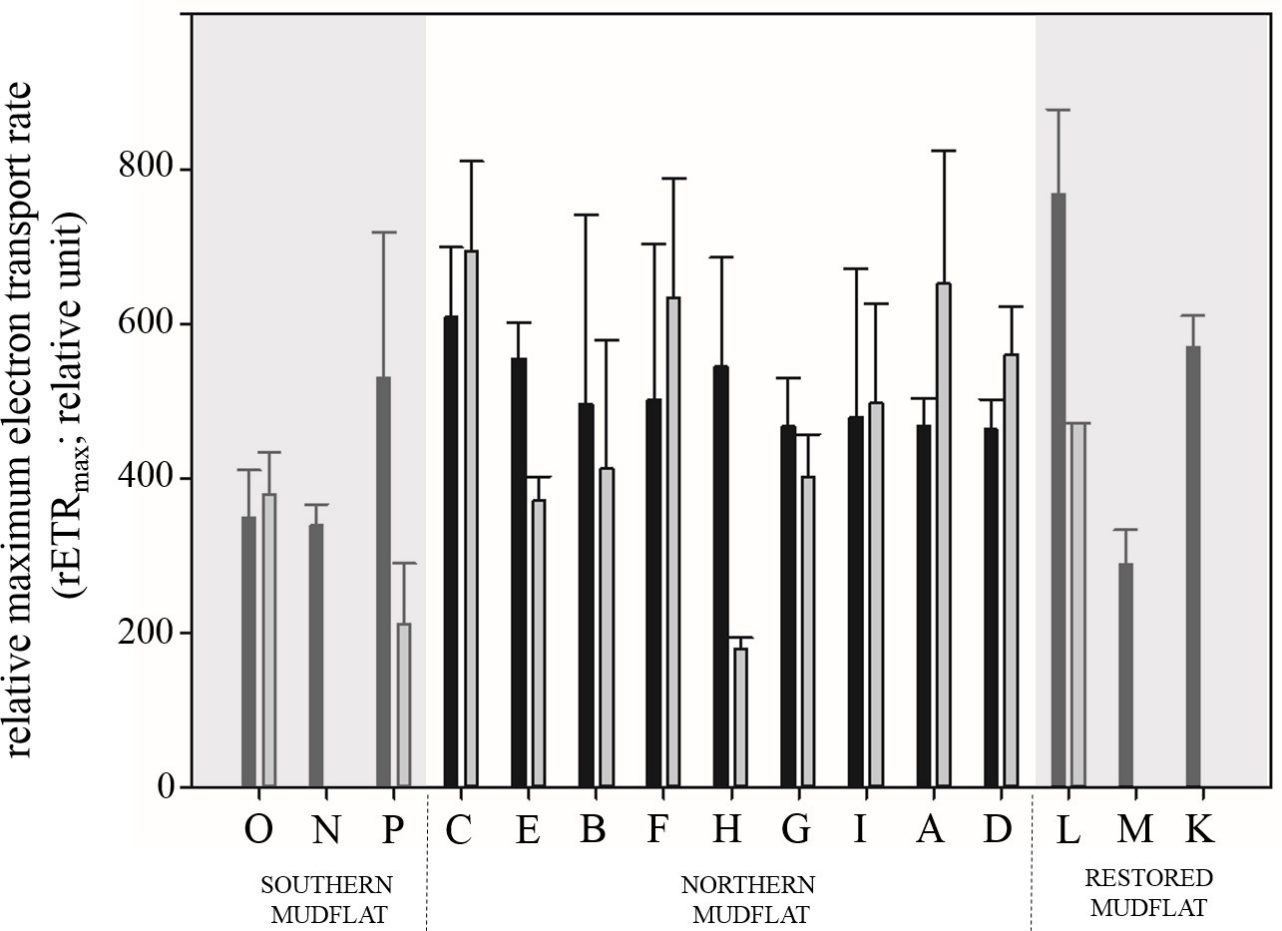
Histogram of relative electron transport rates (rETR_max_; relative unit) for each sampling site in both months. Black bars: values in September; gray bars: values in April.

In September, rETR_max_ (relative unit) values ranged between 288.16 (Site M) and 766.98 (site L) with marked spatial heterogeneity (Fig 9). In April, rETR_max_ values ranged between 179.2 and 693.2 and were significantly lower at the sandiest sites (O, P, N and H) than at the muddiest sites.

The light saturation parameter (E_K_ μmol photons m^-2^ s^-1^) ranged from 431 to more than 3,200 μmol photons m^-2^ s^-1^ (Table 1). Despite high variability among sites, the highest value was recorded at one of the muddiest sites (site L in September) while the lowest value was recorded at one of the sandiest sites (site N in April). We recorded significantly lower E_K_ values in the southern mudflat than in the northern one in April (AOV; p-value < 0.01) and a significant difference between the E_K_ values in the restored mudflat, which were higher than in both the other mudflats in September (AOV; both p-value = 0.06).

The maximum thermal dissipation of excess-absorbed-light energy (NPQ_max_) ranged from 0 to 68.5 (Table 1). NPQ_max_ was significantly lower in April than in September with respective mean values of 1.2 ± 0.9 and 11.1 ± 13. However, the high values observed in September (> 9) were only observed at sandy sites (O, C, N), and at site I. Except at these sites, the mean NPQ_max_ was relatively low with a mean value of 1.6 ± 1.4 in both months. The *n* parameter, corresponding to the degree of sigmoidicity, varied among the sites (Table 1) with values of around 1.0 or slightly below (no sigmoidicity) and values higher than 3.0 (very high sigmoidicity). The biggest differences were found in the light level required for induction of NPQ (indicated by the parameter E_50_) that varied between 150 to values well above growth irradiances (> 5,000 μmol photons m^-2^ s^-1^), especially in September (Table 1). However, the very high values observed in September could easily be due to an estimation bias with too weak RLC irradiance values applied to the samples in September (up to 929 μmol photons m^- 2^ s^-1^) compared to those applied in April (up to 2,309 μmol photons m^- 2^ s^- 1^). Indeed, due to these too low values, the rETR_max_ saturating level was not really reached and model estimation overestimated both values of NPQ_max_ and E_50_ values.

#### Exopolysaccharides

**Fig 10:**
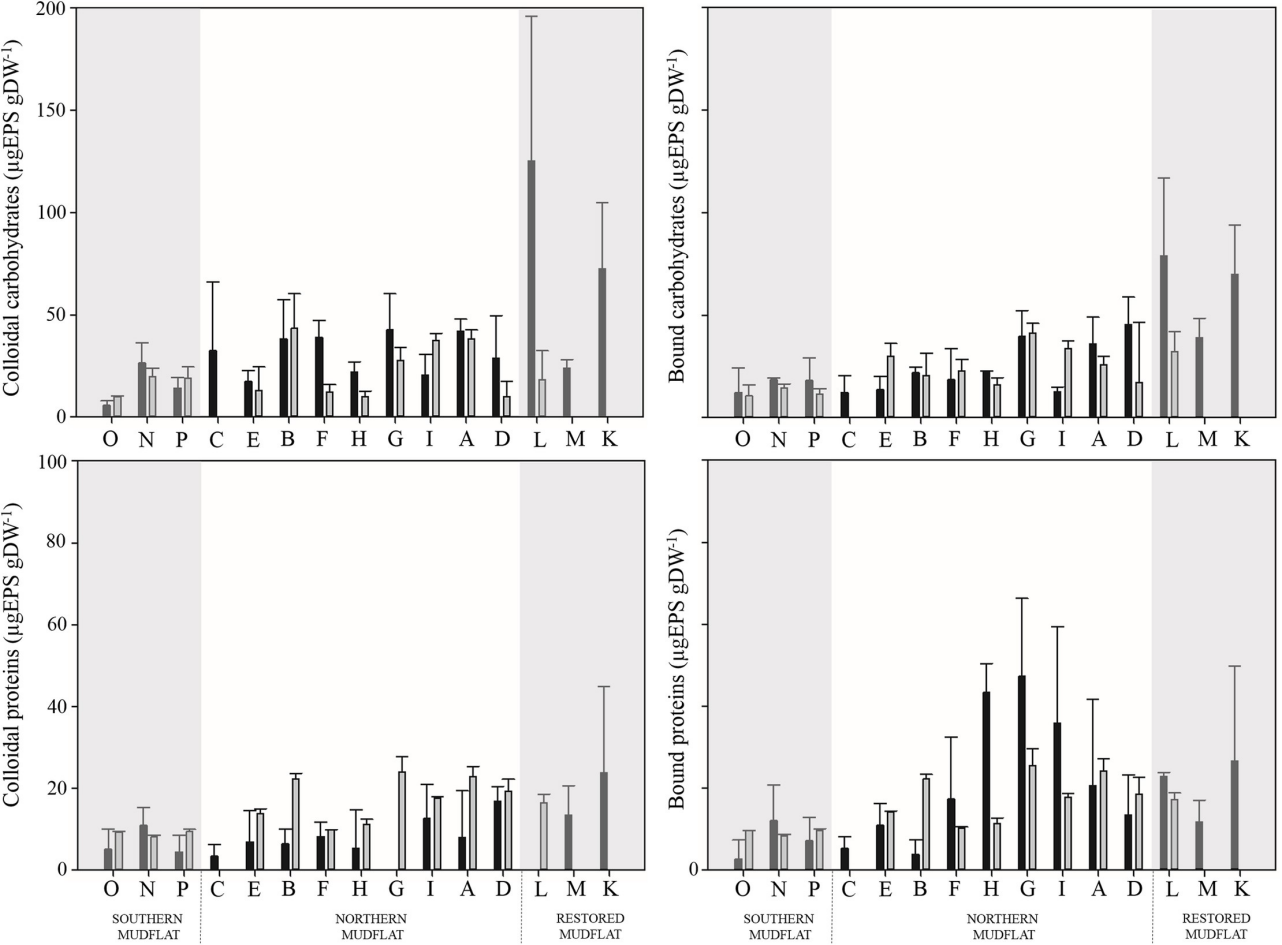
Histogram of exopolysaccharide concentrations (μgEPS gDW^-1^) at each sampling site in both months. With colloidal (on the left) and bound (on the right) for carbohydrates EPS (on the top) and protein EPS (at the bottom). Black bars represent September values and gray bars April values.

EPS were composed of 63.3% of carbohydrates and 36.7% of proteins in equal proportions in the colloidal and bound fractions (Fig 10). In both months and in each of the four fractions of EPS (colloidal and bound proteins or carbohydrates), the concentrations were positively correlated with sediment grain size, and lower EPS concentrations were observed in sandy areas than in muddier ones. Regarding colloidal and bound carbohydrate content, significantly higher concentrations (> 70 μgEPS.gDW^**-1**^) were observed in September in the restored mudflat than in the two others. No significant differences in colloidal protein contents were observed between months whereas higher concentrations (> 35 μgEPS.gDW^**-1**^) of bound proteins were recorded in September than in April at sites G, H and I (characterized by between 51 and 58% of fine particles). Significantly lower colloidal and bound protein contents were observed in the southern mudflat than in the two others. Positive correlations were observed between chl *a* content and concentration of the different fractions of EPS. However, the ratios of EPS per chl *a* unit were higher in April than in September in the southern mudflat with a factor 10 for proteins and 5 for carbohydrates but were relatively similar in both the other mudflats.

#### Relationships between biological and environmental variables

**Fig 11:**
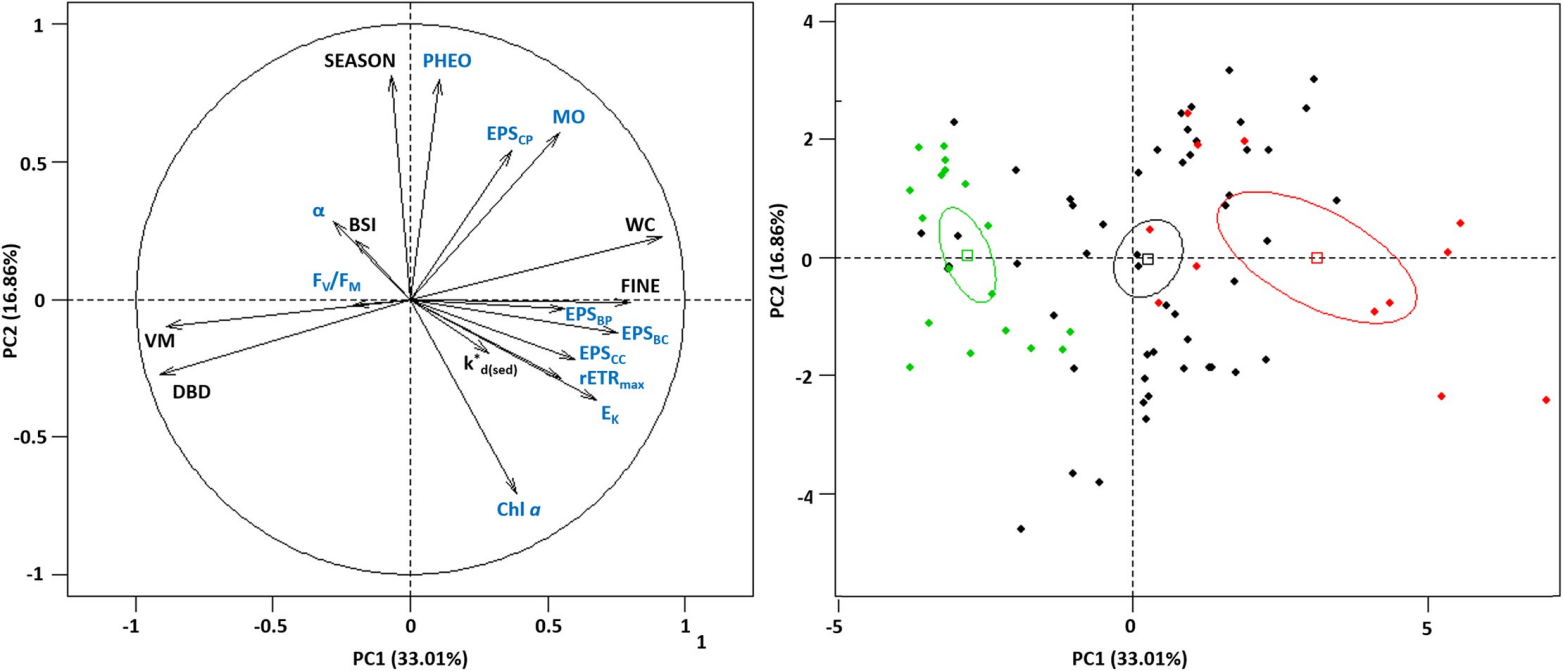
Principal Component Analysis (PCA) of the dataset. Abiotic parameters are in black with the percentage of fine content (FINE); water content (WC), volumetric mass (VM), dry bulk density (DBD), the month number (SEASON; September = 9 and April = 4), the light attenuation coefficient (k*_d(sed)_) and the biofilm structure index (BSI). Biological parameters are in blue with the chlorophyll *a* content (Chl*a*), pheopigment percentage (PHEO), organic matter (MO), photosynthetic parameters (F_V_/F_M_, α, rETR_max_ and E_k_) and exopolysaccharides concentrations (bound (B) and colloidal (C) proteins (P) and carbohydrates (C)). Dimensions 1 and 2 (49.87%) on the left and the ellipses corresponding to each mudflat on the right (the restored mudflat is in red, the northern mudflat in black, and the southern mudflat in green).

Principal component analyses (PCA) were performed on the dataset to explore the relationships between biological and physical parameters (Fig 11A). The 1^st^ and 2^nd^ components explained 49.87% of total inertia while the 3^rd^ component explained 12.32% of total inertia. The 1^st^ principal components (PC1; 33.01% of variance) represented a typical sedimentary axis with the highest variables related to fine particle contents (FINE; 10.74%) and water content (WC; 14.12%) on the right hand side of the axis 1, and the volumetric mass (VM; 13.33%) and the dry bulk density (DBD; 14.01%) on the left hand side of the axis 1. The carbohydrates EPS (EPS_BC_ and EPS_CC_) and the bound protein EPS (EPS_BP_) values were positively correlated with the right side of this axis, while colloidal protein EPS (EPS_CP_) values were positively correlated with the percentage of organic matter (MO). This axis revealed the differences between the sedimentary characteristics of the three mudflats, as confirmed by the ellipses in Fig 11B. The second principal component (PC2; 16.85% of variance) was influenced by the seasonal changes with high contribution of the sampling date (SEASON; 21.70%) and associated variables such as chlorophyll *a* content (Chl *a*; 16.49%), pheopigment percentage (PHEO; 21.06%), and sedimentary organic matter content (MO; 12.07%). The 3^rd^ principal component (PC3; 12.32% of variance) was related to photosynthetic parameters and exhibited the highest contribution for the maximum photosynthetic efficiency (α; 31.57%) and the maximum quantum yield of PSII (F_V_/F_M_; 33.64%).

## Discussion

### Microphytobenthos dynamics in the downstream Seine estuarine mudflats

#### Southern mudflat

The southern intertidal flats (sites O, P and N) can be considered as sandy areas with 22.9% mean fine particle contents (< 63 μm) in September and 5.96% in April, which is explained by dynamics of the river currents [62] and the intensity of seasonal climatic events in this temperate estuary. Site O on the lower foreshore is subject to a stronger current than the two other sites, thus explaining why it has the coarsest sediment. Given the low chlorophyll *a* concentrations (44.8 mg m^**-**^^2^ in September and 5.42 mg m^**-**^^2^ in April) and its vertical distribution, which was relatively homogeneous (Figs 7 and 8) except at site N, which was a little more stratified, this area does not appear to favor the development of microphytobenthic biofilm. The presence of a mussel area close to site N could explain the better stratification of biofilms at this site due to non-trophic interactions [63]. Indeed, reports in the literature show that the presence of mussels can increase nutrient flow, degradation of biodeposits and excretion of bio-compounds [64] as well as modify microphytobenthic communities [65]. Moreover, the high EPS/Chla ratios observed on this mudflat appear to reflect these stressful conditions, particularly high protein excretion. This process, which must be very costly for microphytobenthic cells, could result from cell lysis or be excreted as a strategy against UV radiation or dehydration [66], particularly as epipsammic diatoms cannot migrate vertically [67,68] as a photoprotective strategy in response to excessive light exposure [21,22,41].

Despite a relatively good F_V_/F_M_ (0.65 in September and 0.47 in April), the rETR_max_ were lower than in the other mudflats (this study) with a mean of 464.4 in September and of 315.6 in April. However, NPQ_Ek_ values (0.03 in September and 0.06 in April; Table 1) showed that few photoprotection process were activated during our survey, indicating good photo-acclimation. Moreover, the E_k_ values of 825.1 μmol photons m^-2^ s^-1^ in September and 592.7 μmol photons m^- 2^ s^-1^ in April were high compared to the solar irradiance values measured during exposure, particularly considering the light attenuation in the sediment illustrated by k*_d(sed)_ values.

On the other hand, although it is generally accepted that MPB is rarely nutrient-limited due to its ability to incorporate nutrients from the mineralization of organic matter present in the sediment, nutrient limitation in estuaries can occur during exposure when the pore-water content in surficial sediments decreases (especially in permeable sand). Even though nutrient content was not measured and diatom species were not distinguished in this study, considering the coarseness of the sediment in the southern mudflat and the low contents of organic matter and water (~30%), poor accessibility to nutrients could also explain the difficulty that microphytobenthos face in forming structured biofilms and producing more biomass, particularly since epipsammic diatoms cannot reach nutrients accessible in deeper layers during migration phases [69,70], which applies equally to nitrogen [1,71] and silicates [72,73].

The low chlorophyll *a* concentrations, their vertical distribution and the photosynthetic capacities we measured appear to show that this mainly sandy area of the Seine estuary, which covers 80,000 m^2^, is not the best area for microphytobenthic development and suggests that this sector of the ecosystem cannot strongly support the estuarine trophic networks of the Seine.

#### The restored mudflat

The restored mudflat (sites K, L and M) is a protected area with low tidal flow that is characterized by accumulation of fine particles explaining why each site showed muddy structures with more than 67.3% of fine particles in both months. However, site M, located on the upper foreshore close to vegetated areas presented a lower moisture content (69.4% in September) than the other sites (~200% in September and 106.4% in April).

The chlorophyll *a* concentrations recorded on-site were low, 21.8 mg m^- 2^ in September and 20.7 mg m^-2^ in April. These values could be explained by a potential top-down control by herbivory on MPB biomass [74–76]. Six species of fauna dominate the mudflats in the Seine estuary: *Corophium volutator*, *Macoma balthica*, *Cerastoderma edule*, *Scrobicularia plana*, *Hediste diversicolor and Peringia ulvae* and our study area is mainly dominated by *H*. *diversicolor* [78] whose densities can reach 920 ind m^-2^ [77]. *H*. *diversicolor* is an omnivorous predator [78] that consumes large quantities of microphytobenthic biofilm at the sediment surface [79] during both high and low tide [80]. The high pheopigment contents (degraded chlorophyll compound) measured at these sites support this hypothesis. Moreover, the negative relationship between pheopigment percentages and chl *a* content observed in this study is surely related to the role of grazers on intertidal mudflats [76].

The vertical distribution of chlorophyll *a* in this mudflat was very homogeneous (Figs 7 and 8) and is evidence for the difficulty biofilms face in establishing a stratification with high chl *a* content in surface waters. Although the predation rate may be partly responsible, estuarine particle dynamics could also be involved. Indeed, the mud bedforms and fresh mud deposits in this macrotidal estuary are continuously reworked by intense hydrodynamics, the wave regime, and rain-induced runoff, thereby creating new surface layers that could be a challenge for biofilm formation particularly in winter when hydrodynamics and climate events are strong [81]. In summer, the low river flow could contribute to particle retention in the estuary thereby favoring the deposit of fluid mud, especially in preferential accumulation areas like the restored mudflat [61]. Depending on the thickness of the deposited layer, the MPB cells migrate to build a new pioneering biofilm during a lag phase that is known to last three days in culture conditions [71]. Thereby, the contrast between the length of time needed for biofilm establishment and the frequency with which new sediment layers are deposited on the mudflat could also limit microphytobenthos development. The difficulty that biofilms face in establishing stratification could be heightened by cohesive structure of these muddy habitats that strongly limits light penetration, as illustrated by the values of k*_d(sed)_ and the depth of the euphotic layer [34–37]. Moreover, the high values of carbohydrate EPS that plays a role in the movement of epipelic benthic diatoms and allows the organisms to adhere to sediment surfaces also support this hypothesis and reflect the significant frequency of migration and formation of microphytobenthos biofilms.

These stressful conditions appear to have affected the physiological state of chlorophyll cells in September with low F_V_/F_M_ values (~0.33) and low E_50_/E_k_ values (~0.6) pointing to saturation of photochemistry with significant activation of NPQ. Knowing that the xanthophyll cycle is the main physiological process involved in photoprotection of benthic diatoms undergoing excessive irradiance [21,82] and that this process leads to a decrease in photosynthetic parameters [83], this could explain the low values observed for both F_V_/F_M_ and α. In comparison, in April, site L presented higher values of photosynthetic parameters (F_V_/F_M_ and α) and NPQ processes appeared to less activate when photochemistry was saturated. Nevertheless, rETR_max_ values (~590 μmole^-^ m^-2^ s^-1^ in September and ~467 μmole^-^ m^-2^ s^-1^ in April) were slightly higher in both months than on the southern sandy tidal flats, thereby confirming reports in the literature that affirm that muddy sediments are more suitable habitats for the growth of microphytobenthic diatoms than sandy areas [15,17,84,85]. Moreover, organic content was also higher at the northern sites, which provides a supply of nutrients *via* bacterial remineralization.

Although these observations suggest that the restored mudflat is not the most suitable place for the development of microphytobenthic biofilms, the supposed high consumption activity rates suggest that rapid renewal of MPB strongly supports the food web associated with this mudflat which covers 1,290,000 m^2^.

#### Northern mudflat

The northern mudflat is the widest mudflat of the Seine estuary with an intertidal area of 4,650,000 m^**2**^. The sedimentary structure of this extended area is highly heterogeneous with a percentage of fine particles varying between 21.8 and 58.3% in September and between 10 and 70.1% in April. The lowest values could be explained by erosion rates due to stronger currents in the lower foreshore (site C in September and site H in April) or in the pit (site E in September), but when these three sites are removed the sedimentary structure was rather a mixture of sand and mud with a mean of 58.9 ± 10.2% of fine particles in both months.

With the exception of sandy sites located on the lower foreshore (C in September and H in April), the chlorophyll *a* concentrations measured on this mudflat in the present study were higher than on the other two mudflats in the two months. Evidence for biofilm stratification was clearly visible in the vertical distribution of chlorophyll *a* on this tidal flat (Figs 7 and 8). These observations suggest that the sediment structure composed of sand/mud mixture offer optimum conditions for microphytobenthos growth. This result is important because the general paradigm in the literature states that microphytobenthic growth is higher in mud than in sand [17]. Although this statement is true, sand/mud mixtures (which are less widely studied) could be more suitable habitats for growth of microphytobenthos than both pure sandy or pure muddy area. It can be hypothesized that this result reveals a trade-off between two situations [86]. On the one hand, compared to pure sand, a mud fraction could allow better access to nutrient supplies resulting from the very active remineralization rates of organic matter in the mud. On the other hand, compared to pure mud, a sand fraction would allow light to penetrate deeper layers more easily. Thus, with sufficient access to these two components (nutrients and light), two major sources of variation in microphytobenthos biomass are attenuated. Moreover, sand/mud mixtures probably offer the best habitats thanks to potentially a combination of epipelic and epipsammic benthic diatoms and high biodiversity linked to optimized exploitation of the multiple ecological niches all along the stratified sediment profile. Microphytobenthic biodiversity and species succession thus needs to be studied in such a transient context, particularly in intermediate sediments (sand-mud mixtures). In the present study, investigations were focused on the functional aspects to provide an overview of the link between sediment and microphytobenthos functions (biomass, photosynthesis, EPS excretions), but this emphasizes the fundamental interest of investigating the diversity/functions relationship and the complex functional traits of all these species in more detail.

The parameters related to photosynthetic activity also followed the same pattern, the F_V_/F_M_ values (mean of 0.55 in both season) illustrated the good physiological state of chlorophyll cells, rETR_max_ values were relatively high (mean of 492.15 in both seasons) which is slightly higher than values observed in the restored mudflat and significantly higher than values measured in the southern mudflat (Fig 9). Moreover, the E_50_/E_k_ ratios were higher than in the restored mudflat, indicating that when photochemistry was saturated in terms of electron transport, NPQ processes did not appear to be activated.

The result showing that concentrations of bound proteins were high in the sand/mud mixtures in September emphasizes the functional role that MPB plays in these intermediate sediments, and especially its potential bio-adhesion properties, as also observed in an experimental study in controlled conditions without macrofauna [87]. Thereby, ecosystem resilience should be considerably improved by these features, since EPS secretion by microphytobenthos certainly helps prevent mud erosion in sand/mud mixtures and promotes bed stability.

The northern mudflat, which is characterized by a sand/mud mixture and had the highest chlorophyll *a* biomass, the most sites with stratified biofilms, and good photosynthetic performances, thus appears to be the most suitable place in the downstream Seine estuary for the development of microphytobenthos. With an intertidal area of 4,650,000 m^2^, this mudflat is undoubtedly the most productive area of the estuary whose role in benthic food webs but also in the upper food compartments (fish, birds) is undeniable.

### Impact of human disturbances on the microphytobenthos compartment

The microphytobenthic compartment of the Seine estuary showed high photosynthetic performances with some variations related to specific sediment characteristics. This allows us to hypothesize a major role for this compartment in the primary production pool of the Seine estuary, like in other estuarine systems [1]. However, the productive area (6.02 km^2^ of intertidal mudflat), the light exposure period (tidal regime: 6 hours), and the depth of the euphotic area [46] are strongly limited in mudflats compared with in the water column [88]. Moreover, studies of sediment dynamics in the Seine estuary have revealed a reduction in the extent of mudflat areas due to intense hydrodynamics and anthropogenic disturbances [48]. In this estuary, there is less fluid mud than in the Gironde or the Loire estuaries [89] and the deposition zone is now located in the subtidal areas of the river mouth. Intertidal mudflats are less and less filled by mud inputs and the extent of the mudflats in the downstream estuary has been divided by a factor of 3 since 1975 and by a factor of 5 since 1875. In this context, we can affirm that the quantity of microphytobenthic primary production and contribution of the microphytobenthic compartment compared to that of other primary producers have dramatically suffered from human disturbances. Nevertheless, the fact that, in the present study, the sand-mud mixtures represented the best place for microphytobenthos development could be a positive point to offset the reduction in the extent of the mudflats, strong disturbances linked to human pressure and climate change. Indeed, it appears that the sediment structure in the mudflats of the Seine estuary has evolved from a mainly muddy structure in the 1990s to a predominantly sand/mud mixture over the last decade [48].This intermediate sediment composition appears to be the best habitat in terms of microphytobenthos biomass and photosynthetic performances, as well as bound protein EPS secretion, which helps resist erosion. At the end of this study, a better understanding of the impact of changing ecosystems, including changes in sedimentary structure, on primary biological resources, is now required along with further investigations of microphytobenthic primary production in general and in particular in terms of biodiversity (ecological succession and competition between epipelic and epipsammic diatoms). Our study also highlights the need to deepen our knowledge of the potential role of benthic macrofauna and bacterial loop in microphytobenthos, since at present, all we can do is suggest some connections with the fate of MPB production.
